# Evolution of BA.2.86 to JN.1 reveals that functional changes in non-structural viral proteins are required for fitness of SARS-CoV-2

**DOI:** 10.1128/jvi.00908-25

**Published:** 2025-09-23

**Authors:** Shuhei Tsujino, Masumi Tsuda, Naganori Nao, Kaho Okumura, Lei Wang, Yoshitaka Oda, Yume Mimura, Jingshu Li, Rina Hashimoto, Yasufumi Matsumura, Rigel Suzuki, Saori Suzuki, Kumiko Yoshimatsu, Miki Nagao, Jumpei Ito, Kazuo Takayama, Kei Sato, Keita Matsuno, Tomokazu Tamura, Shinya Tanaka, Takasuke Fukuhara, on behalf of The Genotype to Phenotype Japan (G2P-Japan) Consortium

**Affiliations:** 1 Department of Virology, Faculty of Medicine Sciences, Kyushu University 12923, Fukuoka, Japan; 2 Department of Microbiology and Immunology, Faculty of Medicine, Hokkaido University 12810, Sapporo, Japan; 3 Department of Cancer Pathology, Faculty of Medicine, Hokkaido University 12810, Sapporo, Japan; 4 Institute for Chemical Reaction Design and Discovery (WPI-ICReDD), Hokkaido University 12810, Sapporo, Japan; 5 One Health Research Center, Hokkaido University 12810, Sapporo, Japan; 6 Division of International Research Promotion, International Institute for Zoonosis Control, Hokkaido University 12810, Sapporo, Japan; 7 Institute for Vaccine Research and Development (IVReD), Hokkaido University 12810, Sapporo, Japan; 8 Division of Systems Virology, Department of Microbiology and Immunology, The Institute of Medical Science, The University of Tokyo, Tokyo, Japan; 9 Faculty of Liberal Arts, Sophia University, Tokyo, Japan; 10 Division of Risk Analysis and Management, International Institute for Zoonosis Control, Hokkaido University 12810, Sapporo, Japan; 11 Center for iPS Cell Research and Application (CiRA), Kyoto University 12918, Kyoto, Japan; 12 Department of Synthetic Human Body System, Medical Research Institute, Institute of Integrated Research, Institute of Science Tokyo, Tokyo, Japan; 13 Department of Clinical Laboratory Medicine, Graduate School of Medicine, Kyoto University 12918, Kyoto, Japan; 14 Institute for Genetic Medicine, Hokkaido University 12810, Sapporo, Japan; 15 AMED-CREST, Japan Agency for Medical Research and Development (AMED), Tokyo, Japan; 16 Graduate School of Frontier Sciences, The University of Tokyo 515734 , Tokyo, Japan; 17 Graduate School of Medicine, The University of Tokyo 515734 , Tokyo, Japan; 18 International Research Center for Infectious Diseases. The Institute of Medical Science, The University of Tokyo, Tokyo, Japan; 19 International Vaccine Design Center, The Institute of Medical Science, The University of Tokyo, Tokyo, Japan; 20 Collaboration Unit for Infection, Joint Research Center for Human Retrovirus Infection, Kumamoto University 13205, Kumamoto, Japan; 21 MRC-University of Glasgow Centre for Virus Research 155698, Glasgow, United Kingdom; 22 International Collaboration Unit, International Institute for Zoonosis Control, Hokkaido University 12810, Sapporo, Japan; 23 Laboratory of Virus Control, Research Institute for Microbial Diseases, The University of Osaka 13013, Suita, Japan; Cornell University Baker Institute for Animal Health, Ithaca, New York, USA

**Keywords:** SARS-CoV-2, COVID-19, JN.1, pathogenicity, recombinant virus, non-structural viral protein, S, NSP6, ORF7b

## Abstract

Severe acute respiratory syndrome coronavirus 2 (SARS-CoV-2), the causative agent of coronavirus disease 2019 (COVID-19), is still circulating among humans, leading to the continuous evolution. SARS-CoV-2 Omicron JN.1 evolved from a distinct SARS-CoV-2 lineage, BA.2.86, and spread rapidly worldwide. It is unclear why BA.2.86 did not become dominant and was quickly replaced by JN.1, which possesses one amino acid substitution in the spike protein (S:L455S) and two in the non-spike proteins NSP6 and ORF7b (NSP6:R252K and ORF7b:F19L) compared to BA.2.86. Here, we utilized recombinant viruses to elucidate the impact of these mutations on the virological characteristics of JN.1. We found that the mutation in the spike attenuated viral replication, while the non-spike mutations acted synergistically to enhance replication. This suggests that the mutations in the non-spike proteins compensate for the one in the spike, improving viral fitness, as the mutations in the spike contribute to further immune evasion. Our findings suggest that functional changes in both the spike and non-spike proteins are necessary for the evolution of SARS-CoV-2, enabling evasion of adaptive immunity within the human population while sustaining replication.

**IMPORTANCE:**

Because the spike protein is strongly associated with certain virological properties of SARS-CoV-2, such as immune evasion and infectivity, most previous studies on SARS-CoV-2 variants have focused on spike protein mutations. However, the non-spike proteins also contribute to infectivity, as observed throughout the evolution of Omicron subvariants. In this study, we demonstrate a “trade-off” strategy in SARS-CoV-2 Omicron JN.1 in which the reduced infectivity caused by spike mutation is compensated by non-spike mutations. Our results provide insight into the evolutionary scenario of the emerging virus in the human population.

## INTRODUCTION

Since its emergence in 2019, SARS-CoV-2, the causative agent of coronavirus disease 2019 (COVID-19), has led to a global pandemic. Early SARS-CoV-2 evolved toward increased pathogenicity, but due to its spread and increased human vaccination, Omicron subvariants have diminished pathogenicity and enhanced immune escape compared with ancestral variants (1–10). Importantly, these subvariants continue to circulate in human populations.

Two dramatic events have occurred in the evolution of SARS-CoV-2 during the pandemic. The first was the emergence of Omicron (BA.1) from the Delta subvariant, and the second was the evolution from Omicron BA.2 to BA.2.86 (2, 10). Between Delta and BA.1, 38 mutations were identified in the viral spike (S) protein and 49 in other viral genes. Similarly, BA.2.86 exhibited 32 mutations in S and 14 in other genes compared to BA.2 (Nextstrain; https://nextstrain.org/ncov/gisaid/global/6m) (2, 10). Then, just as BA.2 emerged from BA.1, the descendant JN.1 emerged in the United States in September 2023 and outcompeted BA.2.86 to become the dominant variant (11). As of December 2024, direct descendants of JN.1, including KP.3 and KP.3.1.1, have become predominant globally (12, 13).

Because the JN.1 lineage surged and rapidly outcompeted previously dominant variants in early 2024, the effective reproduction number (R_e_) and immune-evasive properties of the JN.1 variant have been of great interest to researchers, including ourselves (11, 14, 15). JN.1 showed even greater immune evasion than BA.2.86 but exhibited reduced binding affinity for the SARS-CoV-2 receptor angiotensin-converting enzyme 2 (ACE2). Cryo-EM observations revealed that the mutation L455S in the receptor-binding domain (RBD) of the JN.1 S protein disrupted the interaction between RBD and human ACE2 (16). These findings suggest that the lower affinity of the JN.1 vs BA.2.86 S protein for ACE2 impairs viral entry and viral adaptation against host immune defenses. Thus, despite the reduced ACE2 binding affinity of the JN.1 S, the mechanisms underlying its selective advantage and rapid replacement of BA.2.86 remain incompletely understood. In the evolution from BA.2.86 to JN.1, two additional amino acid substitutions—NSP6:R252K and ORF7b:F19L—were acquired in non-spike proteins (https://jbloomlab.github.io/SARS2-mut-fitness/). NSP6 is a multi-spanning transmembrane protein essential for the formation of replication organelles (17). Recent studies have shown that NSP6 can restrict autophagosome expansion and impair lysosome-autophagosome fusion, potentially enhancing viral replication (18). Notably, the R252K mutation in NSP6 has been implicated in enhanced viral RNA replication in single-round infection assay (19). ORF7b is a small transmembrane protein implicated in modulating host responses. It has been shown to disrupt epithelial barrier integrity, induce cell death (20, 21), and localize to the Golgi and endoplasmic reticulum, possibly interfering with intracellular trafficking and innate immune signaling (22). These observations raise the possibility that the acquisition of non-spike mutations may have compensated for the fitness cost associated with reduced receptor binding, thereby contributing to the successful spread of JN.1.

Reverse genetics systems have played a central role in studying viral replication, pathogenicity, and the impact of specific mutations. Since a rapid reverse genetics system for SARS-CoV-2 has been developed (23, 24), we have investigated the viral characteristics of Delta, BA.2, XBB.1.5, EG.5.1, and XEC, elucidating the roles of specific mutations (1, 8, 9, 25, 26). In this study, we used recombinant viruses to examine the differences in viral characteristics between JN.1 and its direct ancestor, BA.2.86. To this end, we analyzed the mutation frequency and identified the mutation(s) that characterize JN.1. Furthermore, we generated recombinant viruses with these mutations and investigated their replication efficiency and intrinsic pathogenicity.

## RESULTS AND DISCUSSION

**Fig 1:**
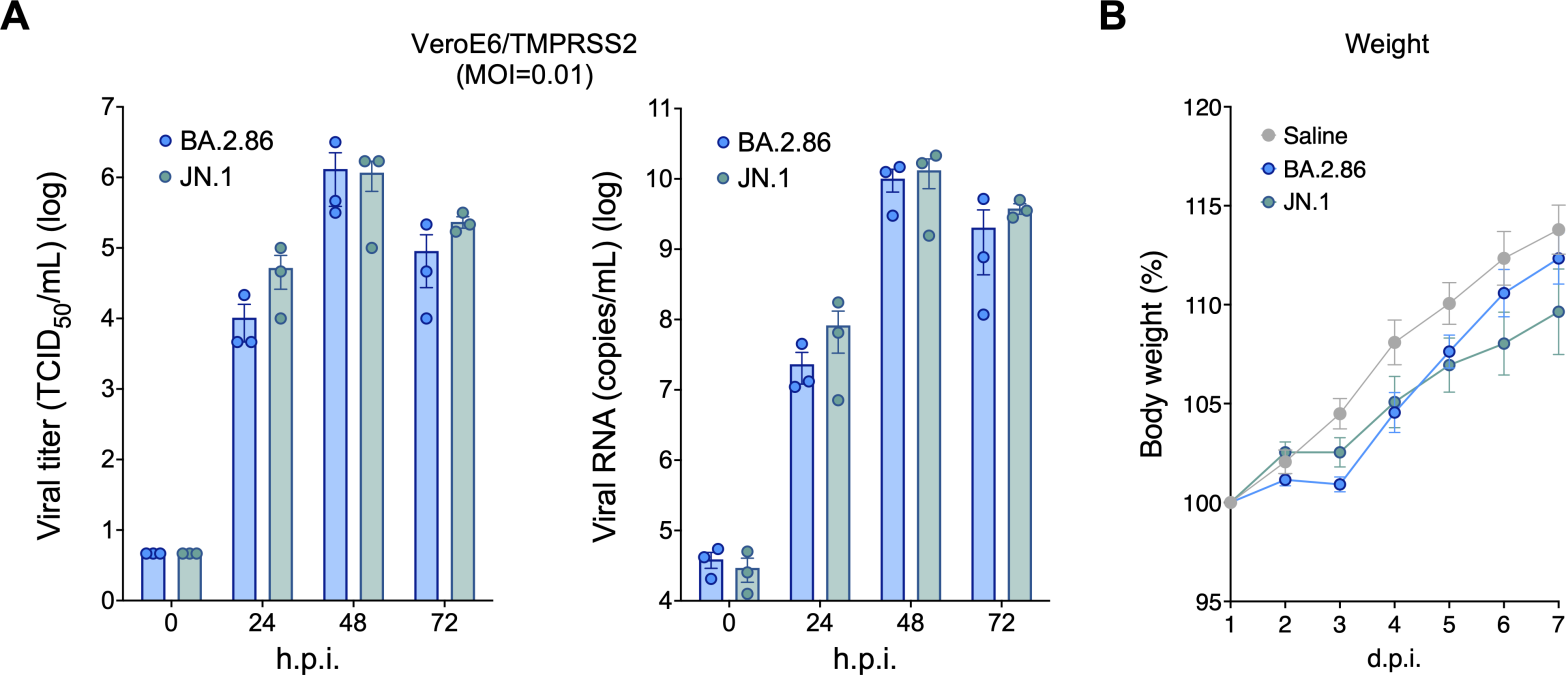
Virological characteristics of SARS-CoV-2 BA.2.86 and JN.1 (**A**) BA.2.86 and JN.1 were used to inoculate VeroE6/TMPRSS2 cells (MOI = 0.01). Viral titers (left) and RNA load (right) of the culture supernatants were quantified at the indicated times post-infection (*n* = 3 independent experiments). (**B**) Syrian hamsters were intranasally inoculated with clinical isolates of BA.2.86 or JN.1 (5,000 TCID_50_) or, as a negative control, saline (each *n* = 6 hamsters of the same age per infection/control group). Error bars are not visible for the viral titer at 0 h.p.i. due to identical replicate values. Data are represented as mean ± SEM. Individual data points are overlaid for bar graphs. Body weight was tracked daily through 7 d.p.i. h.p.i., hours post-infection; d.p.i., days post-infection.

To investigate the replication efficiency and intrinsic pathogenicity, we inoculated VeroE6 cells expressing transmembrane serine protease 2 (TMPRSS2) (27) with clinical isolates of JN.1 (GISAID ID: EPI_ISL_18771637) or BA.2.86 (GISAID ID: EPI_ISL_18233521) (10). In quantifying the infectious viral titers and viral RNA load of the supernatants, we found that the replication properties of BA.2.86 and JN.1 were comparable (Fig. 1A, left and right). Next, we intranasally inoculated hamsters―our established animal model for COVID-19 (1–10)―with either BA.2.86 or JN.1 under anesthesia. The body weights of the hamsters were comparable for the two viruses and, as expected, lower than those of uninfected hamsters (Fig. 1B). These findings suggest that under our experimental conditions, the replication efficiency and intrinsic pathogenicity of JN.1 and BA.2.86 are similar.

**Fig 2:**
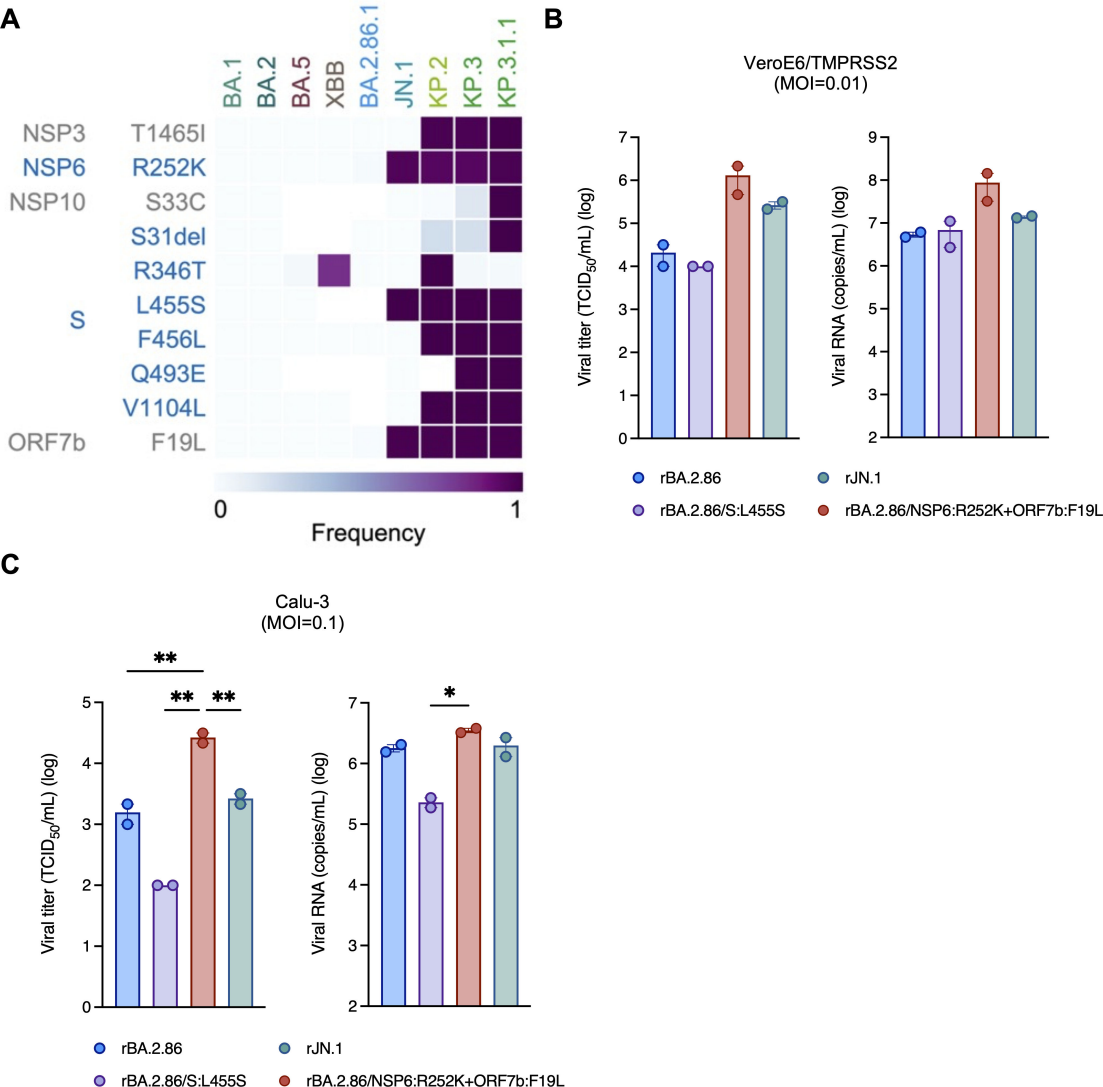
Spike and non-spike mutations define the evolution of BA.2.86 to JN.1 (**A**) Frequency of mutations in BA.1, BA.2, BA.5, XBB, BA.2.86.1, JN.1, KP.2, KP.3, and KP.3.1.1. Only mutations that differ between variants after JN.1 and their parental lineage, BA.2.86, are shown. (**B and C**) Growth of rBA.2.86, rJN.1, rBA.2.86/S:L455S, and rBA.2.86/NSP6:R252K+ORF7b:F19L in the supernatants of VeroE6/TMPRSS2 cells (MOI = 0.01) (**B**) and Calu-3 cells (MOI = 0.1) (**C**) at 24 h.p.i (*n* = 2 independent experiments). Error bars are not visible for the viral titer of rBA.2.86/L455S due to identical replicate values. Data are represented as mean ± SEM. Individual data points are overlaid for bar graphs.

To gain insight into the evolutionary transition from BA.2.86 to JN.1, we examined the frequency of key mutations across multiple Omicron subvariants (Fig. 2A). In BA.2.86, the NSP6:R252K and ORF7b:F19L mutations were present at low frequencies but were subsequently acquired as convergent mutations in JN.1, together with the S:L455S substitution. The JN.1 variant was first detected in September 2023 and became globally dominant by January 2024 (Nextstrain, clade 24A; https://nextstrain.org/ncov/gisaid/global/6m) (11). It then gave rise to several descendant lineages, including KP.2, KP.3, and KP.3.1.1 (Pango nomenclature; https://github.com/cov-lineages/pango-designation) (12, 13, 28). Of note, the three signature mutations originally observed in JN.1—NSP6:R252K, ORF7b:F19L, and S:L455S—have been retained in currently circulating variants, such as KP.3.1.1 (Fig. 2A).

Previous studies, including ours, showed that the S:L455S mutation in JN.1 has a negative effect on RBD-ACE2 binding affinity (11, 16). However, our *in vitro* analysis demonstrated similar growth kinetics for BA.2.86 and JN.1 (Fig. 1A). To further evaluate the impact of spike and non-spike mutations on viral growth in cell culture, we generated recombinant viruses: rBA.2.86; rJN.1; rBA.2.86/NSP6:R252K+ORF7b:F19L (a mutant carrying the two substitutions in NSP6 and ORF7b described above that initially emerged in humans); and rBA.2.86/S:L455S (a mutant carrying the S:L455S mutation).

VeroE6/TMPRSS2 and Calu-3 cells were inoculated with each of the four recombinant viruses. In both cell types, the viral titers and RNA load of rBA.2.86 and rJN.1 were almost identical (Fig. 2B and C), consistent with our results using the clinical isolates. In the supernatants of VeroE6/TMPRSS2 cells, the titers and RNA levels of rBA.2.86/NSP6:R252K+ORF7b:F19L were higher than those of rBA.2.86 (Fig. 2B, left and right). In Calu-3 cells, the titer of rBA.2.86/S:L455S was lower than those of rBA.2.86. On the other hand, the titer of rBA.2.86/NSP6:R252K+ORF7b:F19L was significantly higher than those of rBA.2.86 (Fig. 2C, left). Viral RNA loads also showed a similar trend as well (Fig. 2C, right). These results suggest that the S:L455S mutation decreases replication efficiency, while the NSP6:R252K and ORF7b:F19L mutations increase replication efficiency *in vitro*. Of note, a modest reduction in rBA.2.86/S:L455S replication was observed in Calu-3 cells, but not in VeroE6/TMPRSS2 cells. This difference may reflect the presence of functional antiviral signaling in Calu-3 cells, which is absent in interferon-deficient VeroE6 cells (29, 30). These findings raise the possibility that the replication phenotype associated with the L455S substitution is modulated by cell-intrinsic immune responses.

These mutations in these non-spike proteins in JN.1 may affect replication efficiency. To further investigate this possibility, two additional recombinant JN.1 viruses containing the mutations to revert to the BA.2.86 sequence were generated (rJN.1/NSP6:K252R and rJN.1/ORF7b:L19F). rJN.1, containing an S mutation to revert back to the BA.2.86 sequence, was also evaluated. While designated in these experiments as rJN.1/S:S455L, this virus is the same sequence as rBA.2.86/NSP6:R252K+ORF7b:F19L.

**Fig 3:**
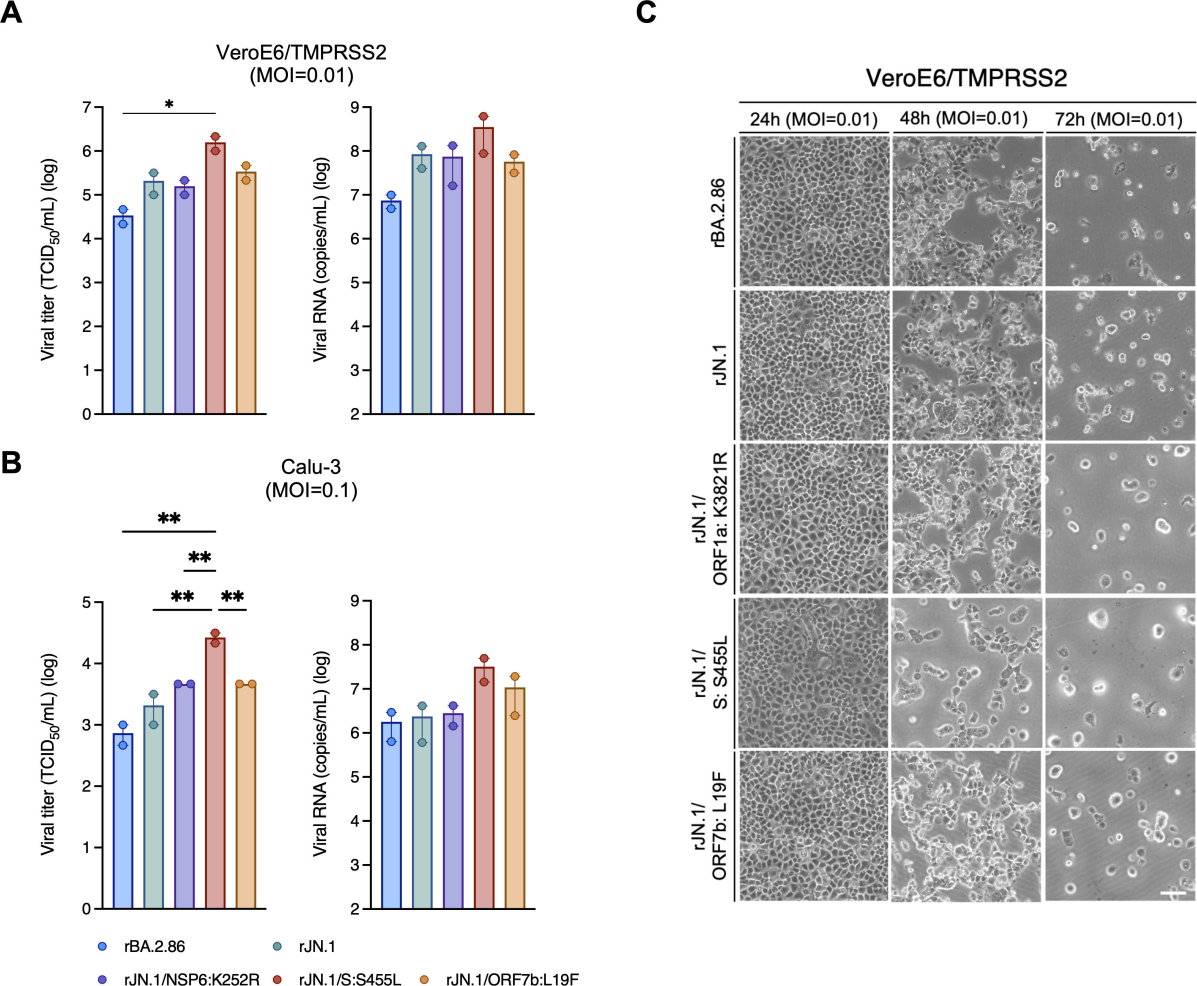
The impact of single mutations on the virological characteristics of JN.1. (**A and B**) Growth of rBA.2.86, rJN.1, rJN.1/S:S455L, rJN.1/NSP6:K252R, and rJN.1/ORF7b:L19F in the supernatants of VeroE6/TMPRSS2 cells (MOI = 0.01) (**A**) and Calu-3 cells (MOI = 0.1) (**B**) at 24 h.p.i (*n* = 2 independent experiments). (**C**) The VeroE6/TMPRSS2 cells were examined by bright field microscopy at the indicated times post-infection to assess cytopathic effect (representative images). Error bars are not visible for the viral titers of rJN.1/NSP6:K252R and rJN.1/ORF7b:L19F in Calu-3 cells due to identical replicate values. Data are represented as mean ± SEM. Individual data points are overlaid for bar graphs. Scale bars, 500 µm.

In VeroE6/TMPRSS2 cells, the growth of rJN.1/S:S455L was significantly higher than those of rBA.2.86, consistent with the greater cytopathic effect observed (Fig. 3A and C). In Calu-3 cells, the titer of rJN.1/S:S455L was significantly higher than those of rBA.2.86 and rJN.1 (Fig. 3B, left). Viral RNA in the supernatants also showed a similar trend (Fig. 3B, right). In both cell lines, the non-spike protein mutants (rJN.1/NSP6:K252R and rJN.1/ORF7b:L19F) did not significantly change growth rate compared to the parental rJN.1. Altogether, these results shown in Fig. 2 suggest that in evolving from BA.2.86 to JN.1, the S:L455S mutation attenuates replication efficiency *in vitro*, while mutations in NSP6 and ORF7b contribute to higher replication efficiency.

**Fig 4:**
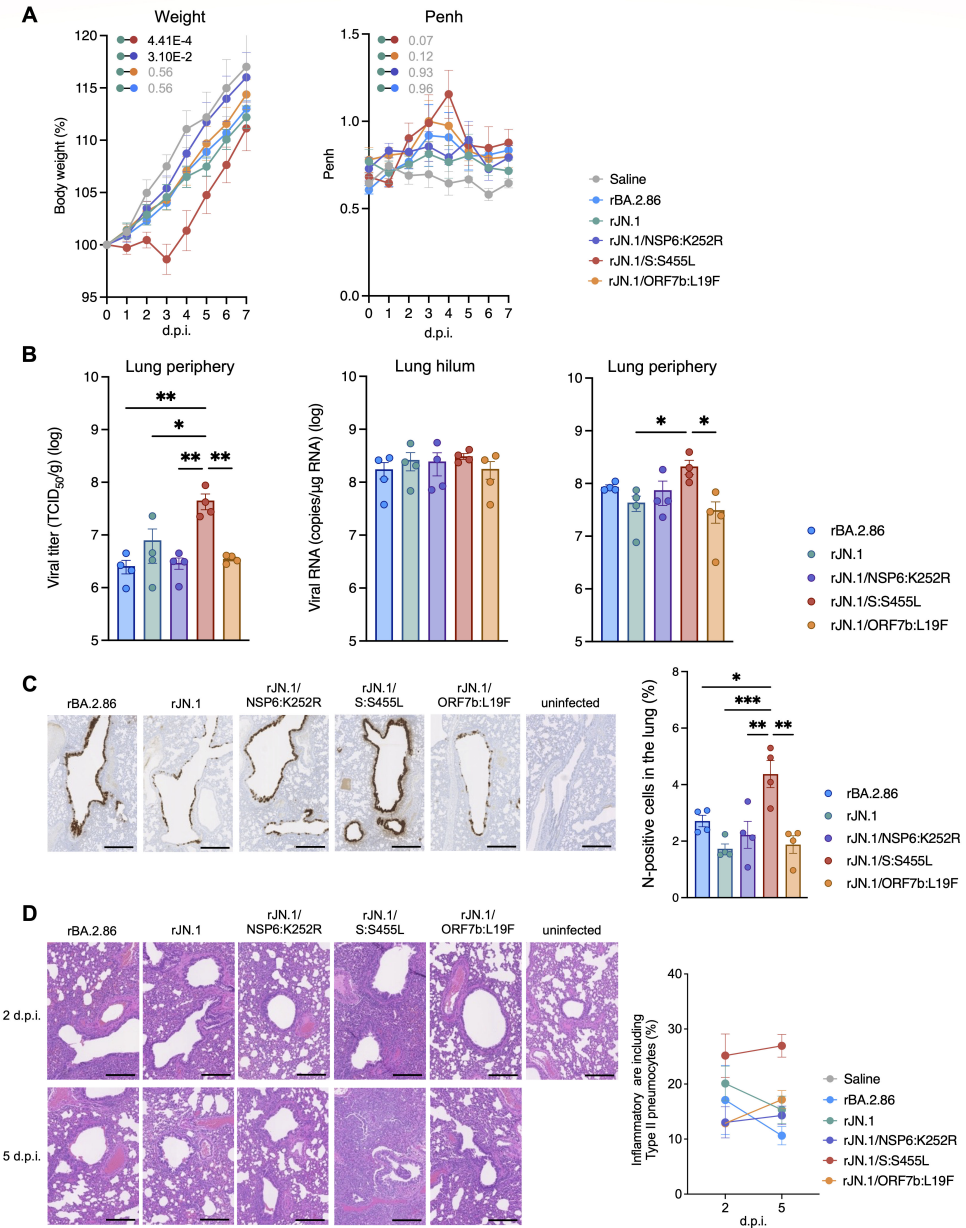
Mutations in non-spike proteins underlie the immunopathogenic features of JN.1. Syrian hamsters were intranasally inoculated with JN.1 backbone viruses (5,000 TCID_50_) or saline (uninfected). (**A**) Body weight (left), enhanced pause (Penh; right) of infected hamsters (*n* = 6 hamsters of the same age per infection/control group). The familywise error rates calculated using the Holm method are indicated in the figures. (**B**) Viral titer in the lung periphery (left), viral RNA load in the lung hilum (middle), and lung periphery (right) of infected hamsters (*n*  =  4 per infection group) at 2 d.p.i. (**C**) Immunohistochemistry of the viral N protein (brown staining) in hamster lung tissue at 2 d.p.i. Representative figures are shown. Scale bars, 500 µm. (**D**) Hematoxylin and eosin staining of the lungs at 2 d.p.i. (upper) and 5 d.p.i. (lower) of infected/control hamsters. Representative figures are shown. Data are represented as mean ± SEM. Individual data points are overlaid for bar graphs. Scale bars, 250 µm.

To investigate the *in vivo* dynamics and pathogenicity of these viruses, Syrian hamsters were intranasally inoculated with rBA.2.86, rJN.1, and the different rJN.1 mutants. Consistent with the *in vitro* findings for the clinical isolates, changes in weight were comparable between hamsters infected with rJN.1 and rBA.2.86. Of the two rJN.1 viruses carrying the single mutation, only rJN.1/S:S455L infection led to significant weight loss compared with the parental rJN.1 infection, which showed the greatest change among all viruses evaluated (Fig. 4A, left). On the other hand, the weight loss of hamsters infected with rJN.1/NSP6:K252R was significantly lower than that of hamsters infected with rJN.1. Hamsters infected with rJN.1/ORF7b:L19F only showed slightly less weight loss compared to hamsters infected with rJN.1. SARS-CoV-2 infection causes a decline in pulmonary function (31), and the degree of deterioration can be used as an index of viral pathogenicity (2). Thus, we assessed the pulmonary function of infected hamsters by measuring enhanced pause (Penh) values. rJN.1/S:S455L infection tended to result in higher Penh values than rJN.1 infection (Fig. 4A, right).

Moreover, to evaluate viral spread in respiratory tissues, we collected the lungs of infected hamsters at 2 and 5 days post-infection (d.p.i.), separating the tissues into the hilum and peripheral regions. In the hilum, the viral RNA load of rJN.1/S:S455L-infected hamsters was comparable to that of rJN.1-infected hamsters (Fig. 4B, middle). In contrast, in the lung periphery region, the viral titer and RNA load of the rJN.1/S:S455L-infected hamsters were significantly higher than those of the rJN.1- and rJN.1/ORF7b:L19F-infected hamsters (Fig. 4B, left and right). At 5 d.p.i., viral titers and RNA load were generally lower across all groups, as expected, and no statistically significant differences were observed among the viruses (Fig. S1). These results suggest that the efficacy of viral spread in the lung is greater with rJN.1/S:S455L than with rJN.1 or rJN.1/ORF7b:L19F.

We also performed immunohistochemistry (IHC) to evaluate the presence of viral N protein in the respiratory tissues of infected hamsters (Fig. 4C; Fig. S2A). In the lung 2 d.p.i., N-positive cells were more strongly detected in the bronchial/bronchiolar epithelia of rJN.1/S:S455L-infected hamsters than in those infected with rBA.2.86, rJN.1, rJN.1/NSP6:K252R, or rJN.1/ORF7b:L19F (Fig. 4C). Then, to evaluate the severity of inflammation upon infection with the mutant viruses, histopathological analyses were performed on the lung tissue (Fig. 4D; Fig. S2B and C). At 2 d.p.i., alveolar damage around the bronchi was prominent in rJN.1/S:S455L-infected hamsters (Fig. 4D). On the other hand, inflammation in bronchi/bronchioles tended to be more limited in rJN.1/NSP6:K252R- and rJN.1/ORF7b:L19F-infected hamsters than in rBA.2.86 and rJN.1. At 5 d.p.i., the alveolar architecture appeared more severely destroyed by alveolar damage and the expansion of type II pneumocytes in rJN.1/S:S455L-infected hamsters (Fig. 4D). No significant differences were found between rBA.2.86-, rJN.1-, rJN.1/NSP6:K252R-, and rJN.1/ORF7b:L19F-infected hamsters. Taken together, these findings suggest that the S:L455S mutation, acquired in the evolution of BA.2.86 to JN.1, attenuates viral growth and pathogenicity.

This study aimed to investigate the virological characteristics of SARS-CoV-2 JN.1 and understand the evolution of this variant from BA.2.86. Our findings suggest that the balance between properties of the spike and non-spike proteins is important for viral fitness and continued circulation of SARS-CoV-2 in humans. Compared with its direct ancestor BA.2.86, JN.1 has three mutations: S:L455S, NSP6:R252K, and ORF7b:F19L (Fig. 2A). Using recombinant mutant viruses generated in both the BA.2.86 and JN.1 backbones, we demonstrated that the S:L455S mutation attenuates replication efficiency and pathogenicity, while the NSP6:R252K and ORF7b:F19L mutations appear to compensate for this attenuation. BA.2.86 did not become predominant in human populations and was quickly replaced by JN.1 (32). This is likely because BA.2.86 exhibited weaker immune evasion than previously dominant variants (33–35). Acquisition of the S:L455S mutation may have helped enhance immune evasion but at the cost of impaired viral replication. Thus, additional mutations in non-spike proteins (NSP6:R252K and ORF7b:F19L), which had already been observed in a minor population of BA.2.86, increased the viral fitness of JN.1 and enabled more efficient circulation in humans. Interestingly, while each single revertant in the JN.1 background (NSP6:K252R or ORF7b:L19F) had little effect on viral replication compared to JN.1 (Fig. 3A and B), the double revertant (rBA.2.86/NSP6:R252K+ORF7b:F19L) in the BA.2.86 background showed increased replication (Fig. 2B and C). These findings indicate that the combined functional effects of the two non-spike mutations acted synergistically, resulting in a phenotypic change that became evident only when both mutations were present. Although direct physical interaction between these proteins is unlikely, combinations of non-spike mutations are known to exert epistatic effects on viral fitness and adaptation (36–38). Such synergy may have contributed to restoring overall viral fitness in JN.1, despite the fitness cost associated with the S:L455S mutation.

Throughout the evolution of Omicron subvariants, SARS-CoV-2 has demonstrated improved immune evasion while maintaining infectivity (39). Acquisition of mutations in the spike protein imposes a weakness on viral fitness. To overcome this, mutations in non-spike proteins could enhance viral fitness by modulating certain virological properties. This “trade-off” strategy has been consistently observed during the circulation of Omicron subvariants in humans. For instance, in BA.1, mutations in both spike and NSP6 were reported to contribute to attenuated pathogenicity (40). In BA.2, we reported that the spike mutation S:L371F enhanced fusogenicity and pathogenicity, while multiple non-spike mutations attenuated replication efficiency and pathogenicity (25). Furthermore, the impairment of major histocompatibility complex suppression due to dysfunctional ORF8 in XBB.1.5 was shown to influence viral pathogenicity (8). Taken together, this knowledge demonstrates that investigating the impact of mutations not only in the spike protein but also in the non-spike proteins is crucial for understanding SARS-CoV-2 evolution.

As we demonstrated for a variety of SARS-CoV-2 Omicron subvariants in the past (2–13, 25, 28), elucidating the virological features of newly emerging SARS-CoV-2 variants is important to determine their potential risk to human society and to understand the evolution of this virus in humans. Accumulating knowledge of the evolutionary traits of newly emerging pathogenic viruses in the human population will be beneficial in preparing for future outbreaks and pandemics.

## MATERIALS AND METHODS

### Cell culture

VeroE6/TMPRSS2 cells (VeroE6 cells stably expressing human TMPRSS2; JCRB Cell Bank, JCRB1819) (20) were maintained in DMEM (low glucose) (Cat#041-29775; FUJIFILM WAKO, Osaka, Japan) containing 10% FBS and 1 mg/mL G418 (Cat#09380-44; Nacalai Tesque, Kyoto, Japan). Calu-3 cells (ATCC, HTB-55) were maintained in Eagle’s minimum essential medium (EMEM) (Cat#056-0838; Sigma-Aldrich, MO, USA) containing 10% FBS and 1% penicillin-streptomycin (Cat#09367-34; Nacalai Tesque).

### Epidemic dynamics analysis and mutation frequency calculations

In this study, we analyzed the viral genomic surveillance data stored in the GISAID database (https://www.gisaid.org; downloaded on 19 May 2025) (41). We used the data collected for SARS-CoV-2 from 1 January 2022 to 1 January 2025 for this analysis. We excluded any data that (i) did not have a collection date and Pango lineage information; (ii) were retrieved from non-human animals; and (iii) were sampled during quarantine. As BA.2.86 has diverged into multiple sublineages, BA.2.86.1 is used in Fig. 2A. Only BA.1, BA.2, BA.5, XBB, BA.2.86.1, JN.1, KP.2, KP.3, and KP.3.1.1 variants are included in Fig. 2A. Additionally, only mutations that differ between JN.1 and their parental lineage, BA.2.86.1, are shown. Mutation frequency of each lineage was calculated by dividing the number of sequences harboring the substitution of interest with the total number of sequences in each lineage.

### Plasmid construction

The nine pmW118 plasmids containing the partial genomes of SARS-CoV-2 BA.2.86 were previously generated (42). To generate the recombinant JN.1 viruses, mutations were introduced by inverse fusion PCR cloning into the plasmids encoding the corresponding BA.2.86 genes. Sequences of all the plasmids used in this study were confirmed by a SeqStudio Genetic Analyzer (Thermo Fisher Scientific, MA, USA) and an outsourced service (Fasmac, Kanagawa, Japan). Primer and plasmid information can be provided upon request.

### SARS-CoV-2 preparation and titration

The working stocks of SARS-CoV-2 virus were prepared and titrated as previously described (8). In this study, stocks were prepared using clinical isolates of BA.2.86 (strain TKYnat15020; GISAID ID: EPI_ISL_18233521) (10) and JN.1 (strain LG0688; GISAID ID: EPI_ISL_18771637).

Recombinant viruses were generated by a circular polymerase extension reaction (CPER) (23). The resultant CPER products were transfected into VeroE6/TMPRSS2 cells as described previously (8). All the viruses were stored at −80°C until use, and viral genome sequences were confirmed by Sanger sequencing (see “Plasmid construction,” above).

### Titration and growth kinetics

The infectious titers of supernatants from infected cell cultures were determined by quantifying the 50% tissue culture infectious dose (TCID_50_) (43). For growth kinetics, VeroE6/TMPRSS2 cells or Calu-3 cells were inoculated with the virus in 12-well plates at a multiplicity of infection (MOI) of 0.01 or 0.1, respectively. The infectious titers of supernatants collected at the indicated time points were then determined.

### Assessment of viral pathogenicity in hamsters

Animal experiments were performed as previously described (1–10). In brief, Syrian hamsters (males, four weeks old) were intranasally inoculated under anesthesia with virus (5,000 TCID_50_ in 100 µL) or saline (100 µL). Body weight was recorded daily until 7 d.p.i. Enhanced pause (Penh) was measured using a Buxco Small Animal Whole Body Plethysmography system (Data Sciences International, MN, USA) every day until 7 d.p.i. Lung tissues were collected at 2 and 5 d.p.i. The viral RNA load in the respiratory tissues was determined by RT-qPCR using a QuantStudio 5 Real-Time PCR system (Thermo Fisher Scientific), as described previously (44, 45). These tissues were also used for immunohistochemistry and hematoxylin and eosin staining as previously described (1–10, 25). Expression of viral proteins was visualized using anti-SARS-CoV-2 N monoclonal antibody (clone 1035111, R&D Systems, 1:400). Images were incorporated as virtual slides by NDP.scan software v3.2.4 (Hamamatsu Photonics, Shizuoka, Japan). The area of N-protein positivity and inflammation was measured using Fiji software v2.2.0 (ImageJ), according to the criteria of certified pathologists (1–10, 25).

### Quantification and statistical analysis

Statistical significance was assessed by one-way ANOVA with Tukey’s multiple comparisons test using GraphPad Prism 10 (GraphPad Software, MA, USA), unless otherwise noted. The values *P* < 0.05 were considered statistically significant (^∗^*P* < 0.05, ^∗∗^*P* < 0.01, ^∗∗∗^*P* < 0.001, ^∗∗∗∗^*P* < 0.0001). In the time-course experiments (Fig. 4A), a multiple regression analysis was performed, including experimental conditions as explanatory variables and timepoints as qualitative control variables, to evaluate the difference between experimental conditions across all timepoints. The initial time point was removed from the analysis. The *P* value was calculated by a two-sided Wald test. Subsequently, familywise error rates (FWERs) were calculated by the Holm method. These analyses were performed in R v4.1.2 (https://www.r-project.org/). All assays were performed independently at least two times.

## Data Availability

The GISAID data sets used in this study are available from the GISAID database (https://www.gisaid.org; EPI-SET-ID: EPI_SET_250604yw). The supplemental tables for the GISAID data sets are available in our GitHub repository (https://github.com/TheSatoLab/JN.1_full).

## SUPPLEMENTAL MATERIAL

Supplemental figures jvi.00908-25-s0001.docx Figures S1 and S2.

ASM does not own the copyrights to Supplemental Material that may be linked to, or accessed through, an article. The authors have granted ASM a non-exclusive, world-wide license to publish the Supplemental Material files. Please contact the corresponding author directly for reuse.
