# Supplementary material for: Evolution of BA.2.86 to JN.1 reveals that functional changes in non-structural viral proteins are required for fitness of SARS-CoV-2
Source: J Virol. 2025 Sep 23;99(10):e00908-25. doi: 10.1128/jvi.00908-25 (PMC12548449; doi:10.1128/jvi.00908-25)
Supplement: Supplemental figures — Figures S1 and S2. [file jvi.00908-25-s0001.docx]

**Fig. S1. Viral RNA quantification and titration in hamster lungs, related to Figures 4B.**

Viral titer in the lung periphery (left), viral RNA load in the lung hilum (middle) and lung periphery (right) of infected hamsters (n = 4 per infection group) at 5 d.p.i. Data are represented as mean ± SEM. Individual data points are overlaid.

**Fig. S2. Pathological features of the infected hamsters, related to Figures 4C and 4D.**

**(A)** Immunohistochemistry of the viral N protein in the lungs of infected hamsters at 2 d.p.i. The percentages of N-positive cells in whole lung lobes are shown in the lower panels for each infection. Scale bars, 5 mm. **(B and C)** Hematoxylin and eosin staining of the lungs of infected hamsters at 2 d.p.i. **(B)** and 5 d.p.i. **(C)** Inflammatory areas with type II pneumocytes are shown in red and the percentage calculated in the lower panels for each infection. Scale bars, 5 mm.


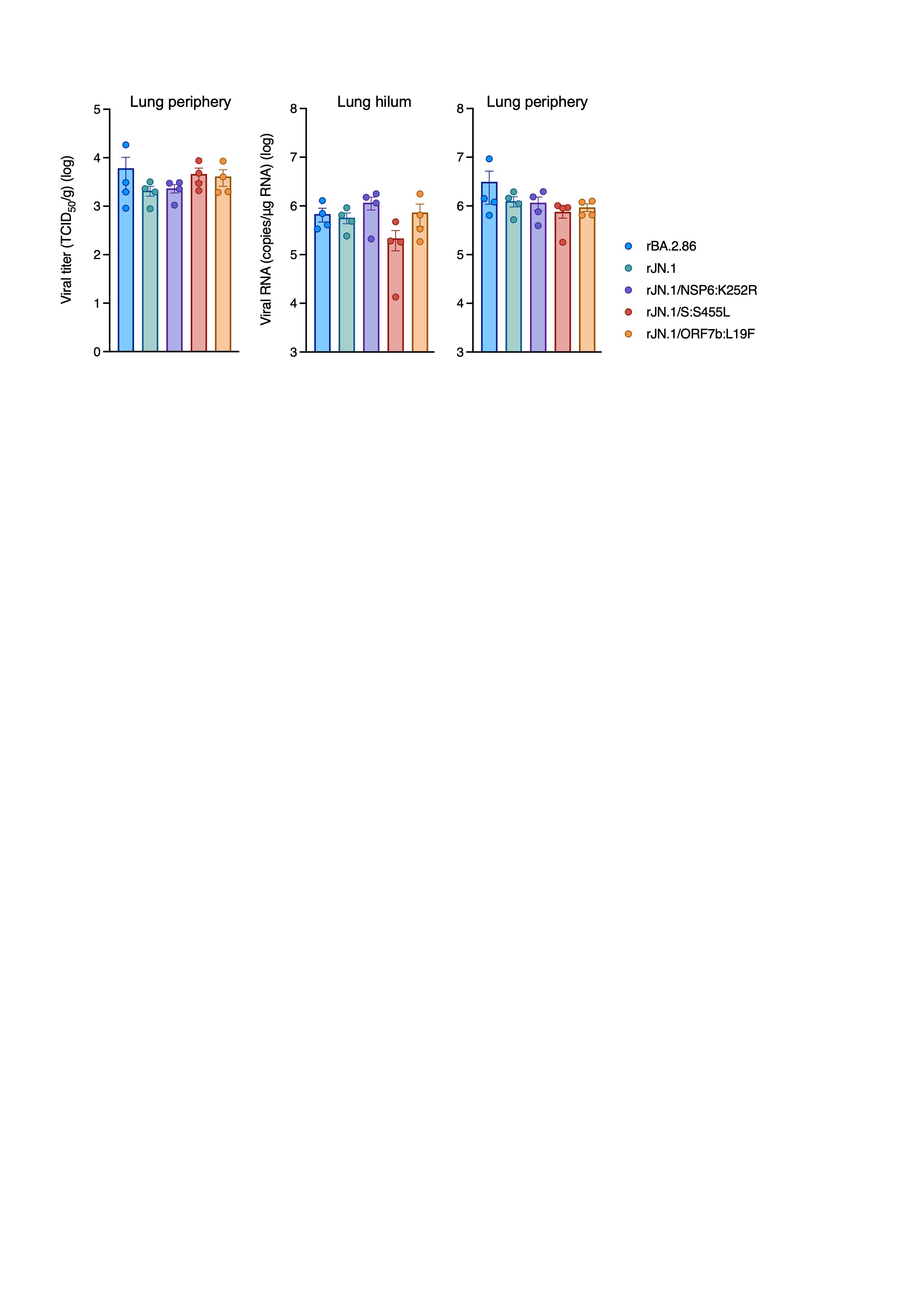


**Fig. S1**


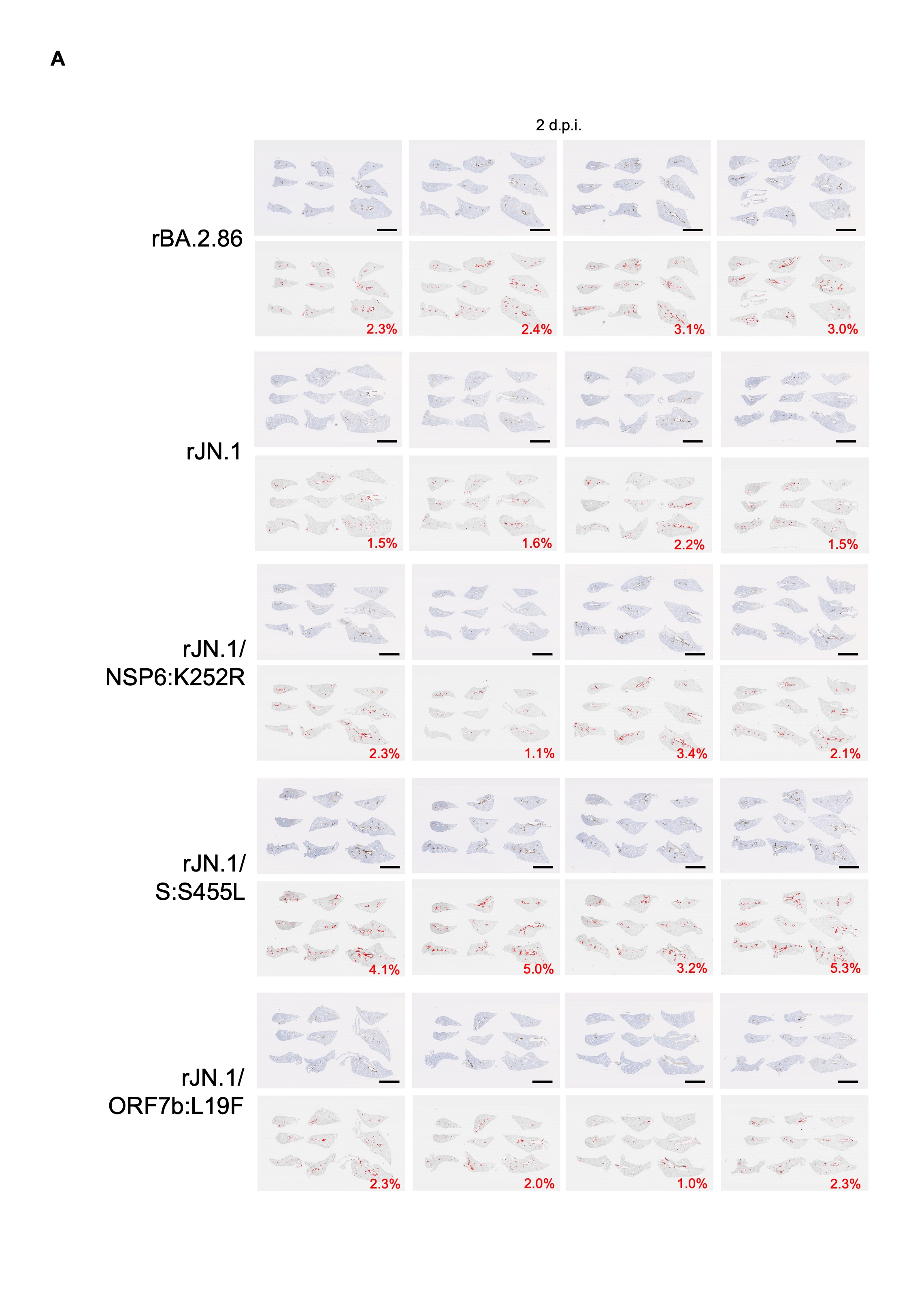


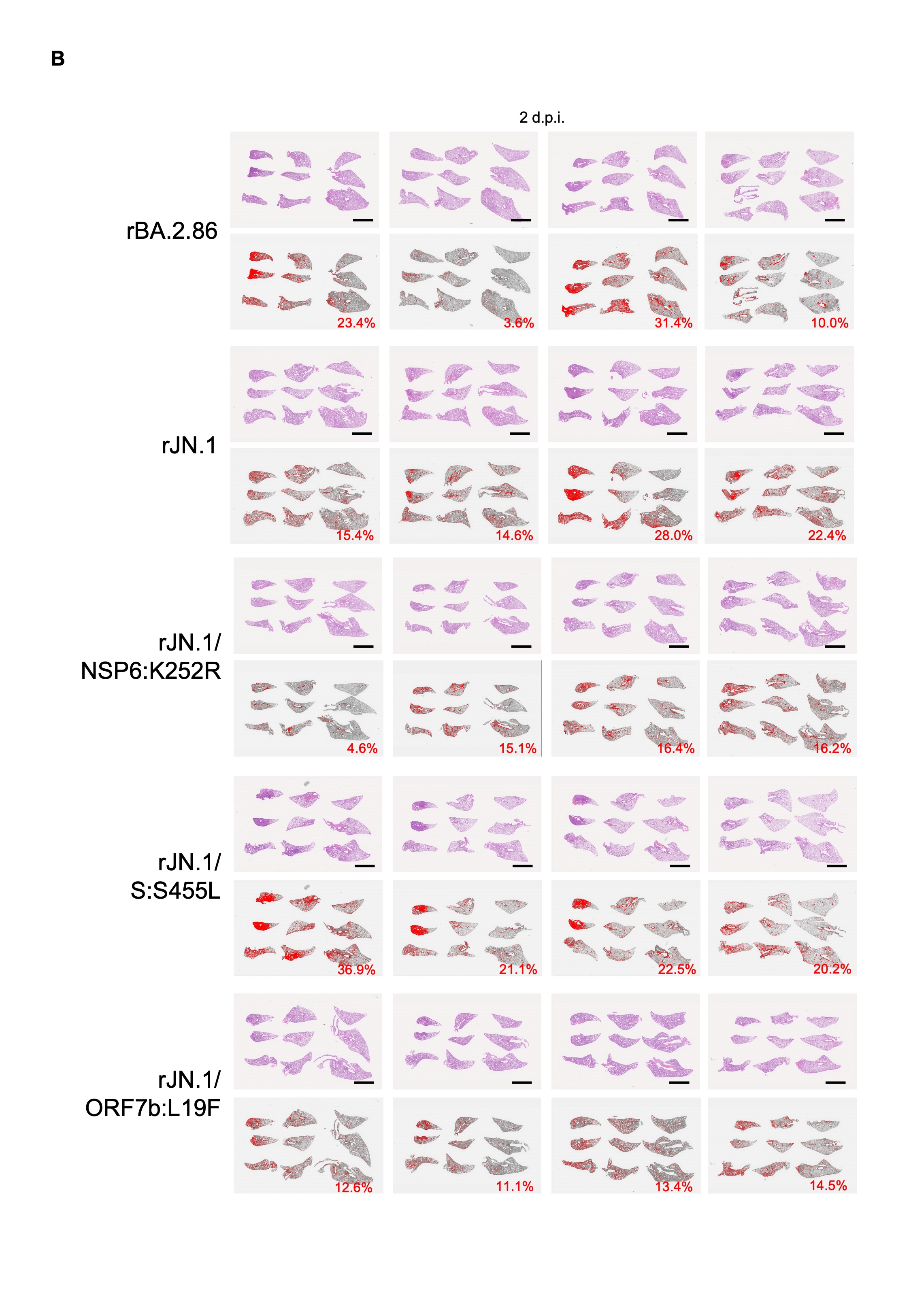


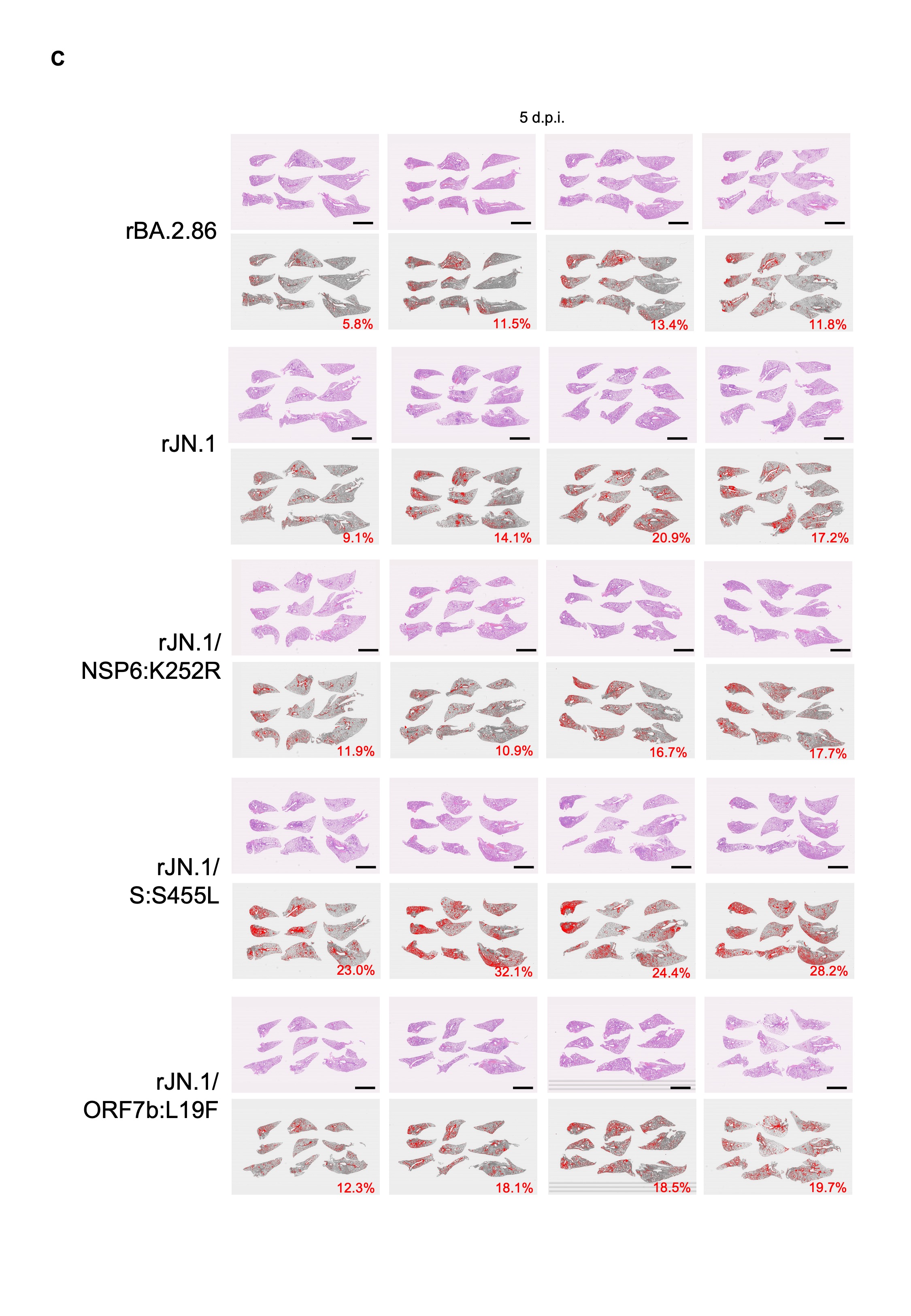


**Fig. S2**
